# Out-of-pocket expenditures associated with double disease burden in Pakistan: a quantile regression analysis

**DOI:** 10.1186/s12889-024-18320-4

**Published:** 2024-03-14

**Authors:** Lubna Naz, Shyamkumar Sriram

**Affiliations:** 1 Department of Economics, School of Economics and Social Sciences, Institute of Business Administration, 75270 Karachi, Pakistan; 2 Department of Social and Public Health, Ohio University, 45701 Athens, OH USA

**Keywords:** Double Disease Burden, Out-of-pocket expenditures, Outpatient Healthcare, Quantile regression

## Abstract

**Background:**

Pakistan is currently experiencing a double burden of disease. Families with members having both communicable and noncommunicable diseases are at a greater risk of impoverishment due to enormous out-of-pocket payments. This study examines the percentile distribution of the determinants of the out-of-pocket expenditure on the double disease burden.

**Method:**

The study extracted a sample of 6,775 households with at least one member experiencing both communicable and noncommunicable diseases from the Household Integrated Economic Survey 2018-19. The dataset is cross-sectional and nationally representative. Quantile regression was used to analyze the association of various socioeconomic factors with the OOP expenditure associated with double disease burden.

**Results:**

Overall, 28.5% of households had double disease in 2018-19. The households with uneducated heads, male heads, outpatient healthcare, patients availing public sector healthcare services, and rural and older members showed a significant association with the prevalence of double disease. The out-of-pocket expenditure was higher for depression, liver and kidney disease, hepatitis, and pneumonia in the upper percentiles. The quantile regression results showed that an increased number of communicable and noncommunicable diseases was associated with higher monthly OOP expenditure in the lower percentiles (10th percentile, coefficient 312, 95% CI: 92–532), and OOP expenditure was less pronounced among the higher percentiles (75th percentile, coefficient 155, 95% CI: 30–270). The households with older members were associated with higher OOP expenditure at higher tails (50th and 75th percentiles) compared to lower (10th and 25th percentiles). Family size was associated with higher OOPE at lower percentiles than higher ones.

**Conclusion:**

The coexistence of communicable and noncommunicable diseases is associated with excessive private healthcare costs in Pakistan. The results call for addressing the variations in financial costs associated with double diseases.

## Background

The double disease burden refers to the simultaneous presence of communicable and noncommunicable diseases among individuals [1]. It has been recognized as one of the major global health challenges [2]. Every year, 56 million people die worldwide, with 38 million (68%) succumbing to noncommunicable diseases. Furthermore, a staggering 16 million (over 40%) deaths occur prematurely, before the age of 70, which is the average life expectancy in the world. Notably, 86% of these premature deaths occur in low-middle-income countries or LMICs [3], highlighting the severity of the burden imposed by NCDs. Moreover, infectious diseases such as malaria, tuberculosis, diarrheal diseases, viral hepatitis, transmitted infections, and neglected tropical diseases (NTDs) remain the leading causes of death in LMICs. These diseases are often called “infectious diseases of poverty” or IDoP, as they disproportionately affect impoverished populations [4], contributing significantly to early-age mortality [5].

However, it is noteworthy that noncommunicable diseases have surpassed infectious diseases as the prime cause of mortality, particularly among the aged and women [6]. Globally, the projected economic impact of cardiovascular disease, diabetes, cancer, and chronic respiratory diseases is overwhelming, with an estimated cumulative output loss of USD 47 trillion throughout 2011–2013. Middle-income countries with expanding populations are expected to bear a substantial proportion of these losses [7].

The double disease burden places significant economic strain on individuals, particularly in LMICs [8]. The financial burden can sometimes lead to compromised adherence to treatment and delays in seeking care, diminishing quality of life, and in severe cases, disability and life-threatening conditions [9]. Eventually, the income forgone while seeking treatment contributes to a decline in economic growth and development [1].

Moreover, the effects of multimorbidity are more pronounced in low-income households, potentially pushing them into poverty or exacerbating existing poverty [10]. Similarly, low-income families, especially those with elderly members or more dependents and without health insurance, are highly vulnerable to financial stress [1].

Evidence suggests that individuals with multimorbidity in noncommunicable diseases incur significantly higher average out-of-pocket expenditures than those with single morbidity, particularly in the case of hospitalization [11]. The adverse effects of multimorbidity in noncommunicable diseases are significant among elderly individuals, females, and urban areas [12].

Wealth status and health insurance are crucial in self-reported diseases [13]. Affluent people have better access to healthcare services and knowledge regarding the implications associated with various diseases [14]. In Bangladesh, India, and Pakistan, older persons with lower wealth status and lacking health insurance are generally at heightened risk of inadequate healthcare utilization [15, 16]. The social protection mechanisms that address healthcare costs can promote equitable access to healthcare services [17].

Pakistan is struggling with multiple challenges in public health, prominently the double disease burden. Approximately 40% of the disease burden comprises communicable infections, encompassing tuberculosis, acute respiratory infections, diarrhea, malaria, hepatitis, and HIV/AIDS. However, over the past few decades, there has been a gradual transition from communicable to noncommunicable diseases (NCDs), such as cardiovascular diseases, diabetes, mental health disorders, cancers, and chronic airway diseases [18]. According to a recent global health report, 60% of the deaths in Pakistan are attributable to NCDs [19].

Pakistan’s healthcare system encompasses both public and private healthcare providers. The private sector caters generally to healthcare needs in urban centers or cities, while the public sector serves in rural regions [16, 20]. However, public sector healthcare spending needs to increase proportionately with the growing population [21]. The National Health Accounts report 2017-18 reveals that the Government of Pakistan contributes to 41% of the total healthcare expenditure, whereas the private sector pays 59%. In private sector healthcare spending, 88% comprises individuals’ out-of-pocket (OOP) expenditures [22]. Moreover, public healthcare delivery is riddled with corruption, nepotism, mismanagement, and negligence [20].

Consequently, most people are forced to choose private healthcare services [23]. However, it burdens people more since private healthcare is an expensive alternative primarily concentrated in urban centers. People living in far-flung areas travel to cities to access medical treatment, which further escalates the overall cost of healthcare, as they need to pay for transport, food, and temporary residence for caretakers [16].

To our knowledge, studies have yet to examine the distribution of private medical care costs or out-of-pocket expenditures associated with double disease burden in the context of South Asian countries. This study contributes to literature in two important ways. First, it focuses on double diseases, which encompass the presence of both communicable and noncommunicable diseases. In contrast, previous research has typically measured multimorbidity based on the presence of one or more communicable diseases or noncommunicable diseases [11, 12, 24]. Second, unlike previous studies that analyzed the effect of multimorbidity on outcome variables using average household out-of-pocket expenditure or population data, this research employs a quantile regression approach to examine the distribution of OOP expenditure associated with double diseases [11]. This approach offers advantages over other empirical models, such as ordinary least squares or generalized linear regression, as it provides coefficients that vary across households or the population [12]. This study used the Household Integrated Economic Survey or HIES 2018-19 and National Health Accounts 2017–2018 for the analysis.

### Data and sample

The study extracted a sample of 6,774 households from the HIES 2018-19 dataset. The selected households reported having at least one member who experienced both communicable and noncommunicable diseases in the three months before the survey. HIES 2018-19 is the eighth survey in a series conducted nationwide by the Pakistan Bureau of Statistics (PBS) in alternating years since 2004. It provides detailed information on 24,809 households related to their sociodemographic characteristics, consumption, income, savings, liabilities, and employment. Information about types of disease, breakdown of OOP expenditure by disease, inpatient and outpatient healthcare utilization, type of healthcare providers, etc., is maintained (HIES 2018-19; section D) under National Health Accounts. Extensive details on the sampling methodology, data collection procedure, and coverage are available at the following PBS website [https://www.pbs.gov.pk/publication/household-integrated-economic-survey-hies-2018-19].

### Outcome variable

This study used out-of-pocket (OOP) expenditure associated with double diseases as an outcome variable. The OOP expenditures comprised expenses on transport, food, doctor fees, hospital admission fees, medicine or vaccine, medical supplies, diagnostic tests, cost of surgery, and accompanying person costs. Since the data were available three months before the survey, this study transformed the OOP expenses to monthly frequency for each household. The adult equivalent OOP expenditures were computed to derive the per-capita measure of the financial burden. Further, the OOP expenditures were adjusted for inflation by using 2015-16 base prices. Finally, the OOP expenditures were translated into US dollars using the average monthly US-Pak exchange rate for 2017-18 [1$: 105 PKR].

### Double disease burden

This study defined double disease burden as “a joint occurrence of both communicable and noncommunicable diseases three months before the survey and the medical treatment the affected individual reported to have received.” The severity of the burden was measured in terms of the number of both communicable and noncommunicable diseases. A discrete variable, comprising at least two and higher values for both communicable and noncommunicable diseases, was used to represent the severity of double diseases.

## Other covariates

Other covariates used in the study included head’s education (no education, primary, secondary, and higher), gender of the head (male or female), marital status (married or unmarried), head’s employment (employed or unemployed), head’s age, healthcare type (inpatient and outpatient), healthcare provider (private and public), co-occurrence of three or more CDs and NCDs (yes or no), household size, older members in household (60 and above), region (rural or urban), province (Punjab, Sindh, Khyber Pakhtunkhwa, Baluchistan) and total household expenditure quantiles (q1: lowest to q5: highest).

### Statistical approach

This study applied the Shapiro Wilk W test on OOP expenditure, and the results showed a skewed nature of the outcome variable, that is, OOP expenditure associated with double disease burden (*p* < 0.05) [25]. Therefore, the quantile regression was employed to assess OOP expenditures across various percentiles, ranging from the 10th to the 90th, for households with double disease [12]. The quantile regression is less affected by the outliers [26]. It allows for estimating the association of OOP expenditures on both communicable and noncommunicable diseases with explanatory variables across the entire range of the outcome variable.

## Results

This study utilized information on all households with members who had experienced both communicable and noncommunicable diseases in the three months before the survey. Of 24,809 households, 23,739 had members who had experienced some illness, of which 39.2% reported to have suffered from one or more communicable diseases only, whereas 32% had experienced one or more noncommunicable diseases. Only 28.5% of households had members with both communicable and noncommunicable diseases. This study used data on 22 diseases, with 12 noncommunicable diseases and 10 communicable diseases. The NCDs included high blood pressure, diabetes, asthma, kidney disease, stroke, muscle pain, cardiovascular disease, brain hemorrhage, cancer, dental care, and ulcers. The communicable diseases included diarrhea, malaria, tuberculosis, hepatitis, chest infection, polio, eye infection, measles, typhoid, pneumonia, and flu/fever. Information unrelated to illness, such as child delivery, family planning, antenatal care, rehabilitative care, and disease undefined or ambiguously defined (‘do not know’), was excluded from the analysis.

Table 1 shows the characteristics of participants with double disease burden. Heads of households with members experiencing the joint occurrence of CDs and NCDs had a mean value 0.12 for higher education, 0.28 for secondary, and 0.15 for the primary education, whereas households with uneducated heads had a mean of 0.43. The mean values for the double burden of diseases was 0.06 for female-headed households, and 0.96 for the male-headed, 0.82 for the employed, 0.07 for unmarried, 0.91 for the outpatient, 0.19 for the public healthcare provider, and 0.35 for the urban. Across provinces, Punjab had the highest mean value for the double disease burden, while Baluchistan showed the lowest value. It may be due to underreporting of the double diseases in Baluchistan, as the area is landlocked with a highly dispersed population and has insignificant access to healthcare facilities. The mean age of a head is 47 years. The mean values of double diseases were higher in large households and those with a higher proportion of older members. The mean values of double diseases were 0.80 and 0.19 for the private and public sectors, respectively. Across rural and urban areas, the rural areas showed a higher mean value of double diseases compared to the urban areas.

**Table 1:** Descriptive statistics of the study variables

Variables	Proportion/mean at CI for CDs	Proportion/mean at 95% for NCDs	Proportion/mean at 95% confidence interval for dual disease
Head education			
No education	0.44[0.436, 0.448]	0.42[0.419, 0.436]	0.43 [0.419, 0.443]
Primary	0.15[0.151, 0.163]	0.15[0.149, 0.161]	0.15[0.147, 0.164]
Secondary	0.27[0.265, 0.278]	0.28[0.274, 0.289]	0.28[0.277,0.299]
Higher secondary	0.12[0.124,0.134]	0.13[0.129, 0.140]	0.12[0.117, 0.132]
Gender-head			
Male	0.91[0.911, 0.920]	0.91[0.905, 0.914]	0.93[0.92, 0.94]
Female	0.08[0.079, 0.088]	0.08[0.085, 0.094]	0.06[0.061,0.73]
Head’s employment			
Unemployed	0.16[0.159, 0.170]	0.19[0.192, 0.206]	0.17[0.166, 0.184]
Employed	0.83[0.829, 0.840]	0.80[0.793, 0.807]	0.82[0.815, 0.833]
Head’s marital status			
Not married	0.087[0.082, 0.091]	0.09[0.087, 0.097]	0.92[0.913, 0.926]
Married	0.91[0.908, 0.917]	0.90[0.902, 0.912]	0.07[0.073,0.086]
Type of healthcare			
Inpatient	0.06[0.058, 0.067]	0.09[0.087, 0.096]	0.08[0.787, 0.092]
Outpatient	0.93[0.932, 0.941]	0.90[0.903, 0.912]	0.91[0.907, 0.921]
Healthcare-provider			
Private	0.70[0.698, 0.712]	0.77[0.770, 0.783]	0.80[0.793, 0.812]
Public	0.29[0.287, 0.301]	0.22[0.216, 0.229]	0.19[0.187, 0.206]
Region			
Rural	0.65[0.646, 0.661]	0.62[0.621, 0.637]	0.64[0.629, 0.652]
Urban	0.34[0.338, 0.353]	0.37[0.362, 0.378]	0.35[0.347, 0.370]
Province			
Punjab	0.46[0.456, 0.472]	0.47[0.465, 0.481]	0.45[0.440, 0.464]
Sindh	0.290[0.283, 0.297]	0.20[0.201, 0.215]	0.25[0.242, 0.262]
KP	0.14[0.136, 0.146]	0.23[0.228, 0.242]	0.20[0.193, 0.212]
Baluchistan	0.10[0.099, 0.108]	0.08[0.201, 0.087]	0.09[0.085, 0.099]
Head’s age	45[45.09, 45.50]	47[46.7, 47.2]	47.7[46.38, 47.03]
Household size	6.5[6.46, 6.56]	6.6[6.59, 6.70]	6.9[6.87, 7.03]
Older member	3.8[3.71, 3.90]	4.5[4.42, 4.64]	4.5[4.41, 4.73]

Authors calculations, HIES 2018-19/NHA 2017-18

**Fig. 1:**
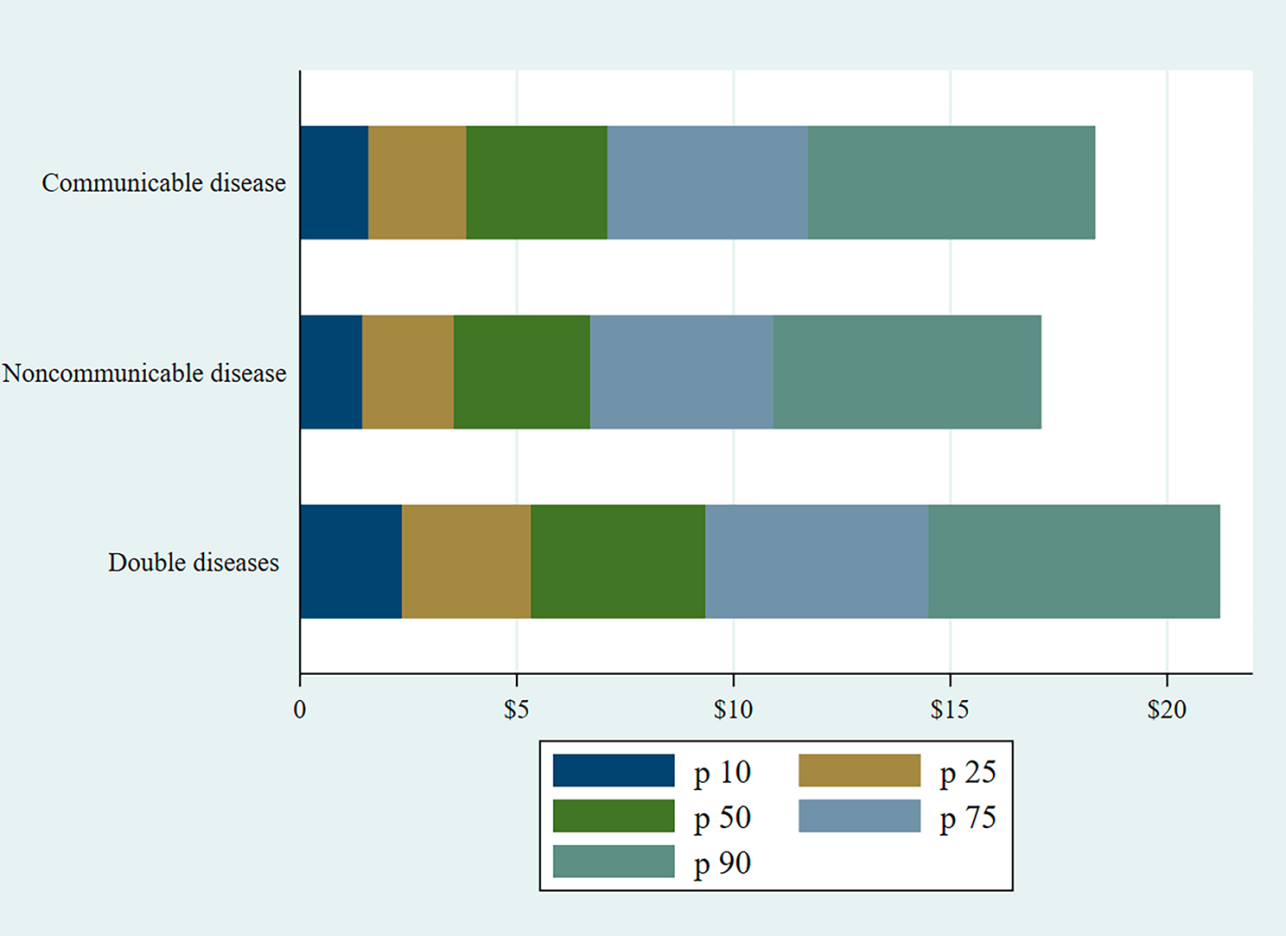
Adult-equivalent OOP expenditures on communicable, noncommunicable, and double diseases across various percentiles in Pakistan

Figure 1 shows the adult equivalent monthly average OOP expenditures on CDs, NCDs, and double diseases across various percentiles. The financial burden of double disease is higher in each percentile compared to communicable only or noncommunicable only, as indicated by PKR 235 (USD 2.23) for double disease, PKR 145 (USD 1.38) for noncommunicable disease, and PKR 158 (USD 1.50) at the 10th percentile in 2017-18, which is not an ordinary cost borne by families given the massive poverty, lack of health insurance, and limited outreach of social protection in Pakistan. Moreover, a family spent on average PKR 1,057 (USD 10) on double disease, PKR 652 (USD 6.21) on noncommunicable disease, and PKR 711 (USD 6.7) on communicable disease at the 50th percentile.

**Fig. 2:**
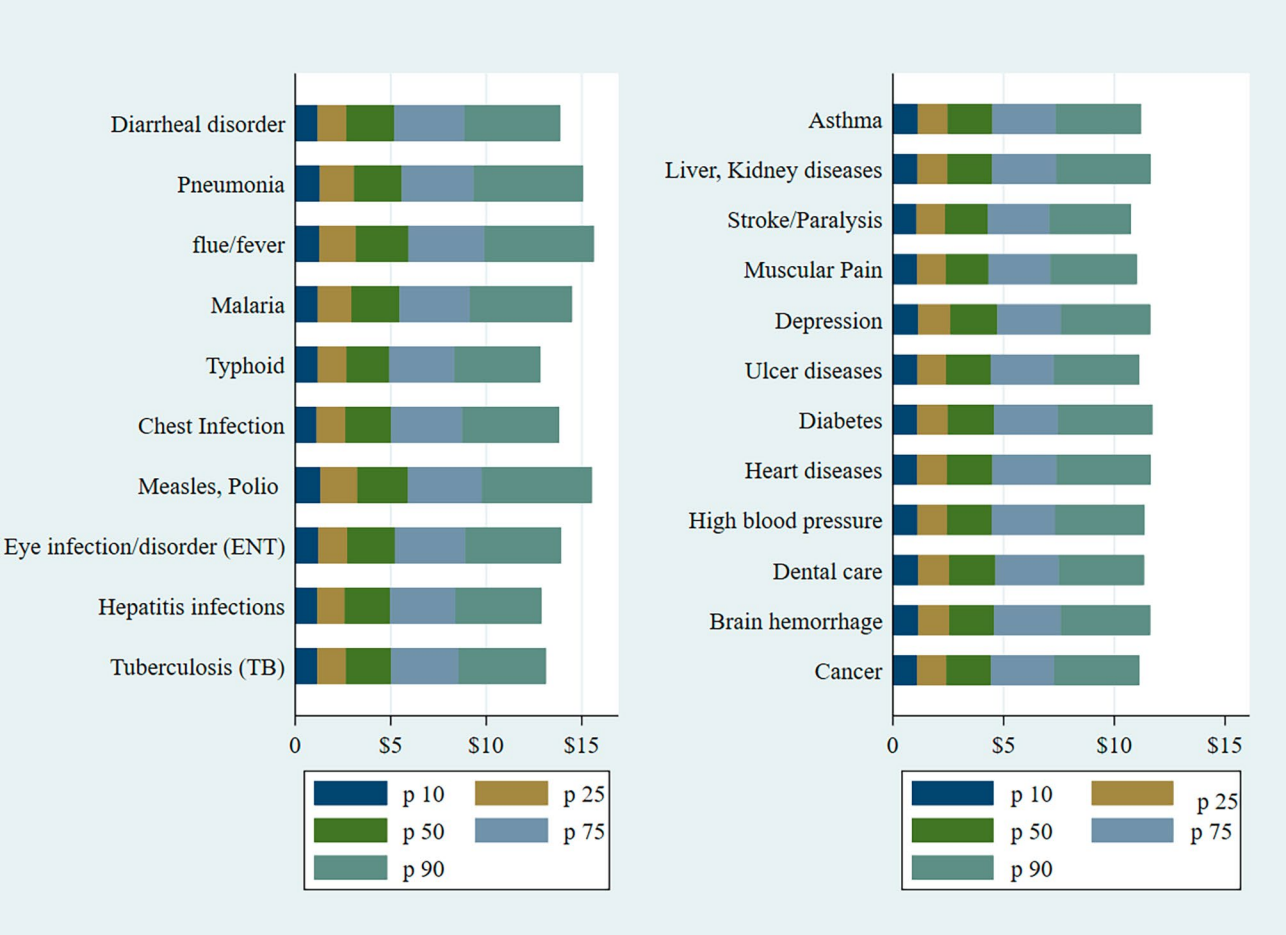
Distribution of adult equivalent OOPE on communicable and noncommunicable diseases across various percentiles

Among all percentiles, households in P90 had the highest OOP expenditures on communicable diseases, as they devoted a significant portion of their spending on measles, flu/fever, pneumonia, and chest infections. households spent more on TB and eye infections in p75. A significant variation is observed across various percentiles for polio, malaria, and hepatitis infection. Among noncommunicable diseases, households spent disproportionally higher on liver or kidney disease, depression, and diabetes, as indicated by p90 (see Fig. 2). Adult-equivalent monthly expenditures on communicable and noncommunicable diseases were primarily evident in p75 and p90.

Table 2 presents the percentage distribution of households and average OOP expenditures across each communicable, noncommunicable, and double diseases for different household expenditure quintiles. The percentages of households with CD, NCD, and double diseases (DDs) were 39.46%, 32.49%, and 28.05%, respectively. The monthly average expenditure on CD, NCD, and double diseases were USD 11.3, USD 21.9, and USD 45.7, respectively. Households in Q1 spent an average of USD 10.9 on CDs, USD 21.3 on NCDs, and USD 39.9 on the double burden of diseases, whereas in Q2, spent an average amount of USD 11.9, USD 23, and USD 43.1 on CDs, NCDs and the double burden of diseases, respectively, in a month. On the other hand, the average out-of-pocket spending in Q5 on CD was USD 14.2, that on NCDs was USD 27.0, and that on double burden disease was USD 55. An increasing trend can be observed in the percentage of households having NCDs and double diseases from Q1 to Q5. Conversely, the percentage of households with CDs declines from Q1 to Q5 (see Table 2).

**Table 2:** Percentage distribution of households and mean OOP expenditures across communicable, noncommunicable, and double diseases by household expenditure quantiles

	Percentage of households with CDs	Communicable disease Mean (CI at 95%)	Percentage of households with NCDs	Non-communicable disease Mean (CI at 95%)	Percentage of households with CD and NCDs	Communicable and non-communicable disease Mean (CI at 95%)
Overall	39.46	$ 11.38 [11.28,11.47]	32.49	$ 21.93 [21.75, 22.11]	28.05	$ 45.75 [45.31, 46.20]
Q1	45.75	$ 10.961 [10.86, 11.05]	29.83	$ 21.31 [21.13, 21.50]	24.42	$ 39.97 [39.44, 40.49]
Q2	41.56	$ 11.94 [11.81, 12.36]	30.96	$ 23.0 [22.76, 23.24]	27.47	$ 43.19 [42.42, 43.96]
Q3	37.62	$ 12.25 [12.36, 12.67]	32.94	$ 23.99 [23.70, 24.28]	29.45	$ 45.13 [44.21, 46.06]
Q4	35.17	$ 13.17 [12.99, 13.35]	34.48	$ 25.20 [24.85, 26.29]	30.35	$ 48.59 [47.51, 49.66]
Q5	32.31	$ 14.21 [13.95, 14.47]	36.62	$ 27.09 [27.40, 27.57]	31.07	$ 55.05 [53.48, 56.62]

Authors calculations from HIES, 2018-19 & NHA data 2017-18; PKR implies Pakistan rupees; PKR was converted into dollars using average of monthly nominal exchange rate for 2017-18 given by the State Bank of Pakistan, as follows, PKR 105: $1

### Results from quantile regression

Table 3 presents the quantile regression results of adult-equivalent out-of-pocket expenditures across the 10th, 25th, 50th, 75th, and 90th percentiles. Among all provinces, Khyber Pakhtunkhwa showed a positive association with OOP expenditures in the lower quantile (p10) and a negative association in the upper quantile (p90). Baluchistan showed a positive and significant association with OOP expenditure on double disease in p25 and p50, whereas a negative association was observed in p90. In contrast, Sindh exhibited a positive association at lower quantiles such as p25 and p50, and a negative association at higher quantiles (p90). The out-of-pocket expenditures were significantly associated with the intensity of the double diseases in each quantile, indicating a higher values at the lower tail compared with the upper tail (coefficient 119.5 for the p10 and coefficient 92.3 for the p90).

**Table 3:** Quantile regression of out-of-pocket expenditures associated with double disease

Outcome variable: Adult-equivalent Monthly OOPE	P10	P25		P50		P75		P90
**Province (RC: Punjab)**								
KP	6.566***	6.254		5.624		-19.04		-46.72*
	(2.274)	(5.003)		(14.66)		(20.84)		(25.75)
Sindh	0.974	2.384		25.69*		1.221		-54.43**
	(2.556)	(3.659)		(15.07)		(18.63)		(21.43)
Baluchistan	4.952	13.19*		47.20**		4.860		-112.0***
	(5.439)	(7.479)		(18.51)		(25.65)		(30.81)
**Number of dual diseases**								
	313.2***	293.6**		258.8***		149.8**		6.053
	(119.5)	(114.9)		(63.99)		(59.52)		(92.36)
**Education (RC: Higher education)**								
No education	0.860	0.657		-3.160		3.996		-6.206
	(3.173)	(4.151)		(12.09)		(14.49)		(23.30)
Primary	-0.288	-2.776		2.566		3.201		21.04
	(2.418)	(3.478)		(13.02)		(22.85)		(26.81)
Secondary	2.955	0.787		16.24		60.20*		98.39***
	(4.473)	(5.766)		(23.64)		(31.22)		(37.87)
**Married head (RC: Unmarried)**								
	-2.475	5.248		33.06		29.36		-33.79
	(5.431)	(7.710)		(25.20)		(40.72)		(38.89)
**Female (RC: Male)**								
	9.816	3.031		2.605		44.85		101.0
	(6.277)	(5.960)		(25.34)		(48.79)		(85.04)
**Employed (RC: unemployed head)**								
	-2.870	-11.03**		-52.91***		-41.72*		-65.61**
	(2.896)	(5.027)		(17.34)		(22.91)		(32.00)
**Head’s age**	-0.860***	-1.436***		-2.417***		-2.424***		-2.759***
	(0.104)	(0.180)		(0.390)		(0.726)		(0.870)
**Older members**	-5.533***	2.615		44.11***		43.37***		45.01**
	(1.731)	(2.674)		(8.739)		(10.51)		(17.72)
**Household Size**	-73.82***	-75.02***		-67.65***		-63.35***		-63.40***
	(1.773)	(2.187)		(2.869)		(3.101)		(3.658)
**Outpatient (RC: In-patient)**								
	-3.525	-2.810		-12.08		6.543		-3.807
	(4.366)	(4.393)		(12.06)		(24.06)		(30.37)
**Public (RC: Private healthcare provider)**								
	-3.734*	-13.55***		-70.73***		-139.1***		-213.4***
	(2.223)	(3.130)		(13.78)		(21.99)		(46.85)
**Rural (RC: Urban)**								
	2.862	4.763*		6.507		-14.46		-33.60
	(2.016)		(2.531)		(11.66)		(15.44)	(24.55)
**Quantiles (RC: Quantile 1)**								
Q2	-15.26***	-27.23***		-94.11***		-89.92***		-155.1***
	(3.415)	(4.442)		(17.65)		(24.79)		(35.42)
Q3	-25.68***	-30.76***		-89.55***		-104.6***		-179.9***
	(3.855)	(5.472)		(17.43)		(33.23)		(35.36)
Q4	-25.59***	-27.23***		-62.58***		-84.71***		-192.5***
	(3.856)	(6.292)		(23.40)		(25.77)		(38.69)
Q5	-25.95***	-14.34**		9.895		26.88		-32.96
	(4.117)	(6.049)		(30.87)		(36.21)		(50.96)
**Constant**	1,499***	1,556***		1,699***		1,804***		2,100***
	(117.6)	(115.7)		(68.39)		(72.61)		(101.2)
**Observations**	6,381	6,381		6,381		6,381		6,381

RC implies reference category; Standard errors in parentheses *** *p* < 0.01, ** *p* < 0.05, * *p* < 0.1

P10, P25, P50, P75 and P90 refer to percentiles

Regarding sociodemographic covariates, the analysis showed an increase in out-of-pocket expenditures with an increase in education level from p10 to p90. Households with older members demonstrated a significantly positive association with OOP expenditures from p50 to p90 but a negative relationship with OOP expenditure at p10. Public healthcare was negatively associated with OOP expenditures across all percentiles. The OOP expenditures on double disease was positively associated with the urban households in p10, p25, and p50, while showed a negative association in p75 and p90.

## Discussion

The findings showed variation in OOP expenditures on double disease across various quantiles (p10, p25, p75 and p90). The joint occurrence of communicable and noncommunicable diseases increases the financial burden on families in lower quintiles, such as p10 and p25, as it requires frequent hospital visits and expenditures on medicines [27]. The results are in agreement with the previous evidence that revealed the concentration of the burden of diseases among households in lower socioeconomic strata. Households from lower quintiles generally cannot afford costly medical treatment [28]. As a result, they are forced to borrow and sell out assets to meet healthcare needs [29]. Furthermore, the financial strain not only adversely affects the quality of life of ailing persons but also make their caretakers face the brunt of the reallocation of time and spending, often pushing families under debt burden and impoverishment [30]. Previous research has indicated OOP expenditures as one of the crucial determinants of poverty [31].

The results showed that OOP expenditures increase with increased education of the head across all percentiles. A similar study suggested that educated people make informed choices regarding healthcare utilization compared to their uneducated counterparts [32]. The lack of knowledge regarding healthcare limits the understanding of complexities associated with diseases, leading to delayed or underutilized medical treatment, and resulting in severe health conditions [33]. The results infer about the positive association of the households with older members and OOP expenditures on double disease are in line with the prior evidence, which indicates that households with elderly members had a higher likelihood of incurring healthcare expenditures than households without elderly members [25].

Compared to the private healthcare, households with double diseases had lower OOP expenditures on public healthcare utilization. The results conform with the evidence [34]. A higher treatment cost for multiple diseases affect the household’s choice of healthcare utilization. Financial barriers may force them to prioritize public healthcare utilization over private services to manage their other needs [35]. Generally, public healthcare services are subsidized in Low-Middle-Income Countries (LMICs). However, the outreach of such services is limited, and the quality is compromised mostly.

The results showed that urban households are associated with higher OOP expenditures than their rural counterparts. The findings align with the earlier evidence [31], which showed that people allocate most of their income to out-of-pocket healthcare payments in urban areas. Expensive medications, healthcare services, and higher living costs in urban areas contribute to excessive OOP expenditures [36]. In our study, the employment, marital status, and age of household heads were found to be negatively associated with OOP expenditures across different percentiles, whereas the strength (magnitude) of the association was significant at the upper tails.

The findings indicate that household size and OOP expenditures on double disease were negatively associated across all percentiles, implying that larger households had lower healthcare spending per adult equivalent compared to smaller families. However, the result contradicts with the prior evidence [37].

This study established that households with female heads were inclined to incur higher out-of-pocket expenditures than male-headed households. It may be due to cultural restrictions on women’s mobility and their networking in Pakistan, which are often helpful in obtaining subsidized medical treatment. A few studies have offered similar insights into the association of the gender of the head with OOP expenditures on multimorbidity [38].

The analysis stresses the need for expanding the outreach of public healthcare, regulating private healthcare, and subsidizing private healthcare costs to deal with the increasing burden of double diseases. Prior evidence supported the efficacy of health insurance, particularly universal health coverage or UHC, and medical subsidies in reducing the financial burden related to double diseases in developing countries [39].

### Strengths and limitations

We employed nationally representative datasets in this study to explore the distribution of the co-occurrence of communicable and noncommunicable diseases and the associated financial burden. A rigorous exploratory analysis was used, including tests for checking skewness, frequency distributions of communicable, noncommunicable, and double diseases, and quantile regression. However, this study has some limitations. For example, the analysis could not distinguish between subsidized or insured medical treatment and medical costs solely borne by households, leading to the likely overestimation of the financial burden of double diseases. National Health Accounts 2017-18 did not provide information on health insurance and subsidized medical care. In addition to this, the data on some diseases, such as AIDS, mental disorders, and skin disease, were not available, which restricted the analysis to selected communicable and noncommunicable diseases. Lastly, due to the unavailability of detailed information, the study could not scrutinize the out-of-pocket expenditures associated with the complex surgical procedures.

## Conclusion

This study provides a comprehensive analysis of OOP expenditures in the context of the double burden of diseases. The findings drawn from the quantile regression showed that the financial burden was unequally distributed across various quantiles. Furthermore, the financial costs associated with double diseases were higher among large-sized families, elderly members, and households with members experiencing certain health conditions, such as depression, liver and kidney disease, hepatitis, and pneumonia.

The higher out-of-pocket medical payments may infer that the population is affluent enough to pay for double diseases. Still, it does not necessarily mean that OOP expenses are a better way to finance medical treatment, especially when socioeconomic gaps are substantial.

The research findings are helpful for policymakers to understand the underlying mechanism for the unequal distribution of OOP expenditures associated with double diseases and develop social security schemes. The government of Pakistan should expand the coverage of subsidized healthcare services to marginalized groups of populations, particularly for those who are suffering from double disease burden. Furthermore, there is a dire need for comprehensive data on illness types, costs associated with illness, healthcare values, and quality of life indicators to provide rigorous evidence for policymaking.

## Data Availability

This article is based on the secondary data obtained from the Pakistan Bureau of Statistics website: https://www.pbs.gov.pk/content/pslm-hies-2018-19-microdata The data is available in the open domain. Anyone can access the HIES 2018-19 data. The National Health Accounts 2017-18 data can be obtained from PBS against payment.

## Abbreviations

AIDS: Immunodeficiency syndrome
CD: Communicable disease
HIES: Household Integrated Economic Survey
NHA: National Health Accounts
NCD: Noncommunicable disease
DDs: Double diseases
IDoP: Infectious disease of Poverty
LMIC: Low-middle-income-countries
OOP: Out-of-pocket
PBS: Pakistan Bureau of Statistics
PKR: Pakistani rupees
USD: United States Dollar
